# Measuring participant rurality in Web-based interventions

**DOI:** 10.1186/1471-2458-7-228

**Published:** 2007-08-31

**Authors:** Brian G Danaher, L Gary Hart, H Garth McKay, Herbert H Severson

**Affiliations:** 1 Oregon Research Institute, 1715 Franklin Boulevard, Eugene, OR 97403 USA; 2 WWAMI Rural Health Research Center, University of Washington, Seattle, WA USA

## Abstract

**Background:**

Web-based health behavior change programs can reach large groups of disparate participants and thus they provide promise of becoming important public health tools. Data on participant rurality can complement other demographic measures to deepen our understanding of the success of these programs. Specifically, analysis of participant rurality can inform recruitment and social marketing efforts, and facilitate the targeting and tailoring of program content. Rurality analysis can also help evaluate the effectiveness of interventions across population groupings.

**Methods:**

We describe how the RUCAs (Rural-Urban Commuting Area Codes) methodology can be used to examine results from two Randomized Controlled Trials of Web-based tobacco cessation programs: the ChewFree.com project for smokeless tobacco cessation and the Smokers' Health Improvement Program (SHIP) project for smoking cessation.

**Results:**

Using RUCAs methodology helped to highlight the extent to which both Web-based interventions reached a substantial percentage of rural participants. The ChewFree program was found to have more rural participation which is consistent with the greater prevalence of smokeless tobacco use in rural settings as well as ChewFree's multifaceted recruitment program that specifically targeted rural settings.

**Conclusion:**

Researchers of Web-based health behavior change programs targeted to the US should routinely include RUCAs as a part of analyzing participant demographics. Researchers in other countries should examine rurality indices germane to their country.

## Background

One of the strengths characterizing Web-based health behavior change programs is the ability to reach participants based in disparate geographic locations. This is particularly important when a set of conditions obtains: (a) when individuals in rural settings are found to have serious modifiable health behaviors; (b) when rural individuals have more limited personal income to pay for personal care and/or transportation to care; and (c) when there are fewer per capita clinic-based health professionals [1]. Although the rural use of the Internet still lags behind usage levels observed for urban areas, Internet usage nonetheless is increasing rapidly [2,3]. In fact, recent data indicates that the adoption rate of broadband Internet services in rural settings is now on parity with urban areas [3]. These trends are encouraging and support using Web-based delivered interventions as one viable approach to reach underserved rural Americans [4]. When describing these interventions, it would be helpful to routinely describe participant rurality in addition to other demographic characteristics. This data can inform recruitment and social marketing efforts, and facilitate the targeting and tailoring of program content.

Fortunately, there is a convenient, empirically-based methodology called RUCAs (Rural-Urban Commuting Area Codes) that can help US researchers accomplish this goal. In this report, we examine the use of RUCAs in two exemplar randomized controlled trials of Web-based tobacco cessation programs: one for smokeless tobacco cessation and the other for smoking cessation to better understand the nature of the population of users for these two intervention trials. We also provide examples of how RUCAs can be used to better understand the reach of behavior change programs delivered via the Internet.

## Methods

### Measuring rurality using RUCAs

There is no one universally agreed upon definition of rurality [5,6]. In their presentation of a handful of different taxonomies for rurality, Hart et al. [7] have recommended use of a flexible, non-monolithic approach to the measurement of rurality that seeks to capture those facets of the urban:rural continuum most relevant to the topic being considered. These researchers describe the development work supported by the University of Washington and the Economic Research Service (with additional funding through the federal Office of Rural Health Policy, Health Resources and Services Administration) that resulted in the development of the 33-category RUCA taxonomy that takes into account both the size of settlement (census-tract data) and the functional relationships between places (tract-level work-commuting data) [8,9]. In an effort to be more usable and comprehensive, RUCA developers subsequently mapped census tract RUCAs onto the US Postal Service ZIP code areas. [10,11] (see Table 1).

**Table 1:** Detailed list of rural urban commuting areas codes

**1 Metropolitan area core: primary flow within an Urbanized Area (UA)**
1.0 No additional code
1.1 Secondary flow 30% through 49% to a larger UA
**2 Metropolitan area high commuting: primary flow 30% or more to a UA**
2.0 No additional code
2.1 Secondary flow 30% through 49% to a larger UA
**3 Metropolitan area low commuting: primary flow 10% through 29% to a UA**
3.0 No additional code
**4 Large rural area core: primary flow within an Urban Cluster (UC) of 10,000 through 49,999 (large UC)**
4.0 No additional code
4.1 Secondary flow 30% through 49% to a UA
4.2 Secondary flow 10% through 29% to a UA
**5 Large rural high commuting: primary flow 30% or more to a large UC**
5.0 No additional code
5.1 Secondary flow 30% through 49% to a UA
5.2 Secondary flow 10% through 29% to a UA
**6 Large rural low commuting: primary flow 10% through 29% to a large UC**
6.0 No additional code
6.1 Secondary flow 10% through 29% to a UA
**7 Small rural town core: primary flow within an Urban Cluster (UC) of 2,500 through 9,999 (small UC)**
7.0 No additional code
7.1 Secondary flow 30% through 49% to a UA
7.2 Secondary flow 30% through 49% to a large UC
7.3 Secondary flow 10% through 29% to a UA
7.4 Secondary flow 10% through 29% to a large UC
**8 Small rural town high commuting: primary flow 30% or more to a small UC**
8.0 No additional code
8.1 Secondary flow 30% through 49% to a UA
8.2 Secondary flow 30% through 49% to a large UC
8.3 Secondary flow 10% through 29% to a UA
8.4 Secondary flow 10% through 29% to a large UC
**9 Small rural town low commuting: primary flow 10% through 29% to a small UC**
9.0 No additional code
9.1 Secondary flow 10% through 29% to a UA
9.2 Secondary flow 10% through 29% to a large UC
**10 Isolated small rural areas: primary flow to a tract outside a UA or UC (including self)**
10.0 No additional code
10.1 Secondary flow 30% through 49% to a UA
10.2 Secondary flow 30% through 49% to a large UC
10.3 Secondary flow 30% through 49% to a small UC
10.4 Secondary flow 10% through 29% to a UA
10.5 Secondary flow 10% through 29% to a large UC
10.6 Secondary flow 10% through 29% to a small UC

Because ZIP codes offer a finer-grained unit of analysis than counties, the ZIP code version of RUCAs is able to differentiate between rural portions of metropolitan counties and urban portions of non-metropolitan counties. RUCAs are now widely used for policy and research purposes [12-15]. Interested researchers can obtain freely-available downloadable copies of the ZIP code version of RUCAs from the University of Washington Rural Health Research Center [10] along with information about ways that RUCAs categories can be aggregated to best facilitate different rurality analyses [10]. One online document on the website highlights the fact that because census-tract data and tract-level work-commuting data change over time – sometimes substantially – it is critical to use the RUCA version that relates to the time when participant ZIP code data was collected and program participation occurred [10]. For example, RUCA version 1.11 is based on commuting patterns for 1990 and the 1998 ZIP code year whereas RUCA version 2.0 is based on commuting patterns for 2000 and the 2004 ZIP code year. The 2006 version of RUCA ZIP code data have recently been released for downloading on the University of Washington website [10].

### RUCA aggregation

We used a common aggregation of RUCA codes that describes locations as one of four types: urban, large rural/town, small rural/town, and isolated small rural/town (see Table 2). Using these groupings, the 2004 US population estimates show that the population is distributed as follows: 81.0% urban, 9.6% large rural, 5.2% small rural, and 4.2% isolated small rural [10]. The three rural groupings can be combined into an aggregate rural group to yield the useful dichotomous description of locations as being either urban or rural. Alternative aggregations of RUCAs could also be considered to more narrowly define groups. For instance, only the 10.0 RUCAs codes could be used to define what might be called "very isolated and small" or the RUCAs could be used in combination with information on paved road travel time to the nearest city [10] to define what might be termed "isolated and remote small" (RUCAs codes 10.0 and 10.2–10.6 which describe 60 minutes or greater from a city).

**Table 2:** RUCAs aggregation

Groups	RUCA codes
Urban	1.0, 1.1, 2.0, 2.1, 3.0, 4.1, 5.1, 7.1, 8.1, 10.1
Large rural/town	4.0, 4.2, 5.0, 5.2, 6.0, 6.1
Small rural/town	7.0, 7.2, 7.3, 7.4, 8.0, 8.2, 8.3, 8.4, 9.0, 9.1, 9.2
Isolated small rural/town	10.0, 10.2, 10.3, 10.4, 10.5, 10.6

### Example 1: Using RUCAs in the ChewFree.com trial

The prevalence of chewing tobacco use is markedly higher in rural counties compared to metropolitan areas [16,17]. Rural chewers wanting assistance to quit have relatively few local resources to help then quit using chewing tobacco [18,19]. The ChewFree.com project, a randomized controlled trial of two Web-based smokeless tobacco cessation websites (an Enhanced condition and a Basic control condition), recruited 2325 participants from across the US during the years 2004–2005. Ethics approval for the research was obtained through the Committee for the Protection of Human Subjects/Institutional Review Board of Oregon Research Institute. All participants provided their informed consent online.

The recruitment methodology, participant exposure to program content, and outcome results are described elsewhere [20-22]. ChewFree participants (2507 of 2523) with valid ZIP codes included in the analysis were drawn from 49 states as well as one individual from the District of Columbia and another from the Armed Forces Pacific.

In addition to the advantages of being available 24×7, Web-based interventions can deliver video content as a way to model the use of behavioral strategies and to provide stories or testimonials that can help build participant motivation to effect a behavior change. In addition, selective use of video when combined with other forms of interactivity may well encourage longer and more frequent visits (building participant engagement and exposure to helpful program content) [20].

Participants who were randomized to the Enhanced Condition (N = 1260) of the ChewFree.com program had the option of reviewing online videos. The default setting for showing video or not was initially determined by an unobtrusive measure of the speed of each participant's Internet connection. In addition, each participant could subsequently override these default settings and change his/her program preferences about viewing the videos. Because successful delivery of video in Web-based programs requires broadband (high-speed) Internet access [23] and the prevalence of broadband use is less in rural settings, we examined the relationship between ChewFree.com participant rurality and their type of Internet access (broadband vs. dial-up). This analysis is limited to participants in the Enhanced condition for whom RUCA categories could be calculated and for whom data on the speed of Internet access was measured at baseline. For purposes of this analysis, we followed the recommendation of Danaher et al. [23] and operationally defined broadband as throughput speeds of at least 384 Kbps.

### Example 2: Using RUCAs in the Smokers' Health Improvement Program (SHIP) trial

In their recent report describing smoking trend data based on the Behavioral Risk Factor Surveillance System (BRFSS), Doescher et al. [24] observed that "...rural residence itself is a risk factor for smoking and that many well-known risk factors for smoking, such as male gender and low socioeconomic status, are especially important among persons residing in rural locations" (p. 115). A growing number of Web-based interventions have focused on helping participants quit smoking [25-29]. The Smokers' Health Improvement Program (SHIP) project used online recruiting methods (Google and Yahoo ad campaigns) to enroll a total of 2318 smokers from the US and Canada to participate in the SHIP randomized controlled trial. Ethics approval for the research was obtained through the Committee for the Protection of Human Subjects/Institutional Review Board of Oregon Research Institute. All participants provided their informed consent online.

For purposes of this analysis, we excluded individuals from Canada and those individuals for whom ZIP code data were invalid or incomplete which yielded the final sample of 2263 smokers drawn from all 50 states and the District of Columbia. Participants were assigned to one of two Web-based programs: (a) the QSN treatment condition which offered a tailored, video-enriched program for preparing to quit, quitting, and maintaining nonsmoking that was derived from a previously-described pilot study [30]; and (b) the Active Lives control condition that provided a personalized physical activity program to help participants become more fit which, in turn, would help them quit smoking.

## Results

Illustrative example data on participant rurality in the Web-based smokeless tobacco cessation trial (ChewFree.com) and the Web-based smoking cessation trial (SHIP) are displayed in Table 3. Owing in large part to large sample sizes and successful randomization, there were no observed differences in rurality between conditions within the ChewFree.com trial (χ^2^(3, *N *= 2507) = 0.55, *p *= .91) and the SHIP trial (χ^2^(3, *N *= 2263) = 0.12, *p *= .99). When we collapsed data across conditions and aggregated RUCA categories to permit an examination of the rural:urban dichotomy, results in both trials indicated that participation differed significantly by rurality: the ChewFree.com trial (χ^2^(1, *N *= 2507) = 192.67, *p *< .001) and the SHIP trial (χ^2^(1, *N *= 2263) = 830.60, *p *< .001).

**Table 3:** Participant rurality in two Web-based tobacco cessation trials

	ChewFree smokeless tobacco cessation trial (N = 2507)		SHIP smoking cessation trial (N = 2263)	
		
RUCA groups	Enhanced Condition	Basic Condition	QSN	Active Lives Control
				
Urban	806 64.3%	795 63.4%	907 80.1%	910 80.5%
Large rural/town	203 16.2%	200 15.9%	119 10.5%	116 10.3%
Small rural/town	146 11.7%	154 12.3%	61 5.4%	58 5.1%
Isolated small rural/town	98 7.8%	105 8.4%	46 4.1%	46 4.1%

In Table 4 we consider participant rurality in each of the two trials using the broader context of the percentage of US population in the same RUCAs groupings [10]. While the rurality of smokers in the SHIP trial closely mirrored the national population distribution, the rurality of smokeless tobacco users in the ChewFree.com trial differed significantly from the population distribution – both when examining all four RUCAs groups presented in Table 4 (χ^2^(3, *N *= 2507) = 511.80, *p *< .001) and also when the data were collapsed into a rural:urban dichotomy (χ^2^(1, *N *= 2507) = 478.50, *p *< .001) with almost twice as many rural participants than would be expected from the distribution of rurality in the US population (36.2% vs. 19.0%, respectively). These Chewfree.com results reflect both smokeless tobacco usage patterns and ChewFree.com's targeted marketing effort.

**Table 4:** Distribution of rural participants in two Web-based tobacco cessation trials and the US population

RUCA groups	ChewFree smokeless tobacco cessation trial	SHIP smoking cessation trial	US population
	(N = 2507)	(N = 2263)	
			
Urban	63.9%	80.3%	81.0%
Large rural/town	16.1%	10.4%	9.6%
Small rural/town	12.0%	5.3%	5.2%
Isolated small rural/town	8.1%	4.1%	4.2%

We also examined type of Internet access and rurality among participants in Enhanced condition in ChewFree.com program. Results (see Table 5) showed roughly equivalent dial-up vs. broadband access among urban participants. In contrast, among rural participants, there was a clear gradient such that dial-up access increased its relative percentage share as the home location became more rural, a result that underscores the importance of taking into consideration the bandwidth constraints of many rural Internet users.

**Table 5:** Rurality by type of Internet access for the Enhanced Condition in the ChewFree.com smokeless tobacco cessation trial

RUCA groups	Dial-Up (N = 722)	Broadband (N = 510)
		
Urban	428 53.8%	367 46.2%
Large rural/town	117 59.1%	81 40.9%
Small rural/town	98 69.0%	44 31.0%
Isolated small rural/town	79 81.4%	18 18.6%

## Discussion

Web-based health behavior change programs are well-suited to reach persons in many settings, including rural locations. Their potential public health impact is particularly important for rural locations wherein access to behavior change services (e.g., tobacco cessation services) is limited by relatively low income, lower rates of health insurance, and the poor availability of health care [24,31]. If the target population is known to be segmented according to rural:urban settings, then describing the study sample according to its RUCA characteristics can facilitate our understanding of the extent to which interventions reached their intended audience [32,33]. RUCAs can also provide valuable feedback to help refine marketing campaigns for Web-based interventions [33]. For example, we found that recruitment to the ChewFree.com research project benefited from using a mixture of both Internet-based marketing and "earned-media" promotions through more traditional media channels (e.g., newspapers and radio) many of which were based in rural settings [21]. In addition, RUCAs can be used to target as well as tailor program content and health messages [34].

Considering tobacco cessation, it is important to note that although Doescher et al. [24] recently reported the rural vs. urban distribution of smoking behavior by counties, to date we do not have finer-grained data that describes the number of smokers or smokeless tobacco users by ZIP codes. Thus, for tobacco cessation and very likely for many other important health behaviors, RUCAs do not yet provide us with the denominator to describe the percentage of eligible participants reached by Web-based tobacco cessation interventions [35]. Nonetheless, ZIP code-based RUCAs datasets for age, race, sex, population, and income (planned), and travel distance time to cities with a population of 50,000 or more can also be used to fine-tune the research. Likewise, additional analyses could combine RUCAs with other geocoded datasets such as the ZIP code-based Consumer Health Profiles available through the Office of Cancer Communications of the National Cancer Institute [36,37], proprietary market segmentation datasets from Claritas, Inc[38,39], and US Census Bureau data on defined region, division, and state [40]. Finally, it is helpful to consider ways that RUCAs can mesh with other geography-based tools such as spatial analysis [41].

One noteworthy limitation of the current report is that it uses a taxonomy that applies only to US locations. Not only do US-based Web interventions attract participation from other countries [e.g., [27]], but Web-based interventions obviously can be hosted in and marketed to participants in countries other than the US. In this regard, other countries have their own measures for categorizing rurality that could potentially be usefully employed to describe participation in their Web-based programs. Examples include the use of Rural and Small Town (RST) designations as a part of the Metropolitan Area and Census Agglomeration influenced Zones (MIZ) in Canada [42,43], the Accessibility/Remoteness Index (ARIA+) in Australia [44], and the Carstairs area deprivation measures in the UK [45,46].

## Conclusion

Because RUCAs linked to ZIP code data can be downloaded at no cost, there appear to be few barriers to obtaining the considerable benefits associated with routinely reporting RUCA-measured rurality along with other key indices of participant demographics in reporting results of Web-based health behavior change programs targeted to US participants. Using data drawn from two Web-based RCTs we highlighted examples showing ways that RUCAs can be used as an integral evaluation component.

## Competing interests

The author(s) declare that they have no competing interests.

## Authors' contributions

BGD conceived of the study, conceptualized ideas, supervised its conduct, and designed and coordinated the data collection phase. LGH, HGM, and HHS all helped to interpret findings and contributed to the text. All authors reviewed drafts of the manuscript and approved the version to be published.

## Pre-publication history

The pre-publication history for this paper can be accessed here:



## References

[B1] DeLeon PH, Wakefield M, Hagglund KJ, Stamm BH (2003). The behavioral health care needs of rural communities in the 21st century. Rural behavioral health care: An interdisciplinary guide.

[B2] Horrigan JB (2006). Home broadband adoption 2006. http://www.pewinternet.org/pdfs/PIP_Broadband_trends2006.pdf.

[B3] Bell P, Reddy P, Rainie L (2004). Rural areas and the Internet: Rural Americans' Internet use has grown, but they continue to lag behind others. http://www.pewinternet.org/pdfs/PIP_Rural_Report.pdf.

[B4] Stamm BH, Stamm BH (2003). Bridging the rural-urban divide with telehealth and telemedicine. Rural behavioral health care: An interdisciplinary guide.

[B5] Rickets TC, Johnson-Webb KD, Taylor P (1998). Definitions of rural: A handbook for health policy makers and researchers. http://www.shepscenter.unc.edu/research_programs/rural_program/ruralit.pdf.

[B6] Hewitt M (1989). Defining rural areas: Impact on health care policy and research. http://govinfo.library.unt.edu/ota/Ota_2/DATA/1989/8912.PDF.

[B7] Hart LG, Larson EH, Lishner DM (2005). Rural definitions for health policy and research. Am J Public Health.

[B8] Morrill R, Cromartie J, Hart LG (1999). Metropolitan, urban, and rural commuting areas: toward a better depiction of the US settlement system. Urban Geography.

[B9] Hart LG (2006). Use of RUCAs in health services research.. http://depts.washington.edu/uwrhrc/uploads/AcadHeal06Y008.pdf.

[B10] RHRC (2006). Rural-urban commuting area codes (version 2.0). http://depts.washington.edu/uwruca/.

[B11] WSDOH (2006). Guidelines for using rural-urban classification systems for public health assessment. http://www.doh.wa.gov/Data/Guidelines/RuralUrban.htm.

[B12] Rosenblatt RA, Andrilla CH, Curtin T, Hart LG (2006). Shortages of medical personnel at community health centers: implications for planned expansion. JAMA.

[B13] Baldwin LM, MacLehose RF, Hart LG, Beaver SK, Every N, Chan L (2004). Quality of care for acute myocardial infarction in rural and urban US hospitals. J Rural Health.

[B14] Evans TC, Wick KH, Brock DM, Schaad DC, Ballweg R (2006). Academic degrees and clinical practice characteristics: The University of Washington physician assistant program: 1969-2000. J Rural Health.

[B15] Chan L, Hart LG, Goodman DC (2006). Geographic access to health care for rural Medicare beneficiaries. J Rural Health.

[B16] USDHHS (2005). Results from the 2004 National Survey on Drug Use and Health.

[B17] Howard-Pitney B, Winkleby MA (2002). Chewing tobacco: who uses and who quits? Findings from NHANES III, 1988-1994. National Health and Nutrition Examination Survey III. Am J Public Health.

[B18] Novotny TE, Pierce JP, Fiore MC, Davis RM (1989). Smokeless tobacco use in the United States: The adult use of tobacco surveys. NCI Monogr.

[B19] Severson HH, Eakin EG, Lichtenstein E, Stevens VJ (1990). The inside scoop on the stuff called snuff: An interview study of 94 adult male smokeless tobacco users. J Subst Abuse.

[B20] Danaher BG, Boles SB, Akers L, Gordon JS, Severson HH (2006). Defining participant exposure measures in Web-based health behavior change programs. J Med Internet Res.

[B21] Gordon JS, Akers L, Severson HH, Danaher BG, Boles SM (2006). Successful participant recruitment strategies for an online smokeless tobacco cessation program. Nicotine Tob Res.

[B22] Severson HH, Gordon JA, Danaher BG, Akers L (2007). ChewFree.com: Evaluation of a Web-based cessation program for smokeless tobacco users. Nicotine Tob Res.

[B23] Danaher BG, Jazdzewski SA, McKay HG, Hudson CR (2005). Bandwidth constraints to using video and other rich media in behavior change websites. J Med Internet Res.

[B24] Doescher MP, Jackson JE, Jerant A, Hart LG (2006). Prevalence and trends in smoking: A national rural study. J Rural Health.

[B25] Lenert L, Muñoz RF, Stoddard J, Delucchi K, Bansod A, Skoczen S, Perez-Stable EJ (2003). Design and pilot evaluation of an internet smoking cessation program. J Am Med Inform Assoc.

[B26] Lenert L, Muñoz RF, Perez JE, Bansod A (2004). Automated e-mail messaging as a tool for improving quit rates in an Internet smoking cessation intervention. J Am Med Inform Assoc.

[B27] Muñoz RF, Lenert LL, Delucchi K, Stoddard J, Perez JE, Penilla C, Perez-Stable EJ (2006). Toward evidence-based Internet interventions: A Spanish/English Web site for international smoking cessation trials. Nicotine Tob Res.

[B28] Etter JF (2006). The Internet and the industrial revolution in smoking cessation counselling. Drug Alcohol Rev.

[B29] Walters ST, Wright JA, Shegog R (2006). A review of computer and Internet-based interventions for smoking behavior. Addict Behav.

[B30] Feil EG, Noell J, Lichtenstein E, Boles SM, McKay HG (2003). Evaluation of an Internet-based smoking cessation program: Lessons learned from a pilot study. Nicotine Tob Res.

[B31] CASA (2000). No place to hide: Substance abuse in mid-size cities and rural America. http://www.casacolumbia.org/absolutenm/articlefiles/379-no_place_to_hide_01-28-00.pdf.

[B32] Glasgow RE, Vogt TM, Boles SM (1999). Evaluating the public health impact of health promotion interventions: The RE-AIM framework. Am J Public Health.

[B33] Maibach EW, Van Duyn MA, Bloodgood B (2006). A marketing perspective on disseminating evidence-based approaches to disease prevention and health promotion. Prev Chronic Dis.

[B34] Kreuter MW, Lukwago SN, Bucholtz RD, Clark EM, Sanders-Thompson V (2003). Achieving cultural appropriateness in health promotion programs: targeted and tailored approaches. Health Educ Behav.

[B35] Glasgow RE, Klesges LM, Dzewaltowski DA, Bull SS, Estabrooks P (2004). The future of health behavior change research: What is needed to improve translation of research into health promotion practice?. Ann Behav Med.

[B36] NCI (2006). Consumer health profiles: A proprietary database of health behavior, demographic, lifestyle, and geographic data for audience segmentation. http://cis.nci.nih.gov/research/CHP_FACT_SHEET.pdf.

[B37] Miner JW, White A, Lubenow AE, Palmer S (2005). Geocoding and social marketing in Alabama's cancer prevention programs. Prev Chronic Dis.

[B38] Claritas (2005). PRIZM NE Profile Analysis: Widget Corporation. http://www.claritas.com/collateral/segmentation/corp-col-2030-0605.pdf.

[B39] Claritas (2006). PRIZN NE: The new evolution in segmentation. http://www.claritas.com/claritas/Default.jsp?ci=3&si=4&pn=prizmne.

[B40] Bureau USC (2006). Federal Information Processing Standards (FIPS) Codes. http://www.census.gov/geo/www/fips/fips.html.

[B41] RHRC (2006). Census Division Nine: Pacific. http://depts.washington.edu/uwruca/Map_4_divisions.html.

[B42] CIHI (2006). Canada's rural communities: An assessment of their health status and health determinants. http://secure.cihi.ca/cihiweb/dispPage.jsp?cw_page=GR_1529_E.

[B43] du Plessis V, Beshiri R, Bollman RD (2001). Definitions of rural. Rural Small Town Can Anal Bull.

[B44] GISCA (2006). About ARIA+ (Accessibility/Remoteness Index of Australia). http://www.gisca.adelaide.edu.au/products_services/ariav2_about.html.

[B45] Walters K, Breeze E, Wilkinson P, Price GM, Bulpitt CJ, Fletcher A (2004). Local area deprivation and urban-rural differences in anxiety and depression among people older than 75 years in Britain. Am J Public Health.

[B46] Levin KA, Leyland AH (2006). Urban-rural inequalities in ischemic heart disease in Scotland, 1981-1999. Am J Public Health.

